# Targeting neuroendocrine abnormalities in Parkinson’s disease with exercise

**DOI:** 10.3389/fnins.2023.1228444

**Published:** 2023-09-08

**Authors:** Nijee S. Luthra, Demetra D. Christou, Angela Clow, Daniel M. Corcos

**Affiliations:** ^1^Department of Neurology, University of California, San Francisco, San Francisco, CA, United States; ^2^Department of Applied Physiology and Kinesiology, College of Health and Human Performance, University of Florida, Gainesville, FL, United States; ^3^Department of Psychology, School of Social Sciences, University of Westminster, London, United Kingdom; ^4^Department of Physical Therapy and Human Movement Sciences, Feinberg School of Medicine, McCormick School of Engineering, Northwestern University, Chicago, IL, United States

**Keywords:** Parkinson’s disease, exercise, neuroendocrine, biomarkers, neurohormone

## Abstract

Parkinson’s Disease (PD) is a prevalent and complex age-related neurodegenerative condition for which there are no disease-modifying treatments currently available. The pathophysiological process underlying PD remains incompletely understood but increasing evidence points to multiple system dysfunction. Interestingly, the past decade has produced evidence that exercise not only reduces signs and symptoms of PD but is also potentially neuroprotective. Characterizing the mechanistic pathways that are triggered by exercise and lead to positive outcomes will improve understanding of how to counter disease progression and symptomatology. In this review, we highlight how exercise regulates the neuroendocrine system, whose primary role is to respond to stress, maintain homeostasis and improve resilience to aging. We focus on a group of hormones – cortisol, melatonin, insulin, klotho, and vitamin D – that have been shown to associate with various non-motor symptoms of PD, such as mood, cognition, and sleep/circadian rhythm disorder. These hormones may represent important biomarkers to track in clinical trials evaluating effects of exercise in PD with the aim of providing evidence that patients can exert some behavioral-induced control over their disease.

## 1. Introduction

Parkinson’s disease (PD) is a neurodegenerative disorder involving progressive loss of nigrostriatal dopaminergic neurons. While motor symptoms such as tremor, bradykinesia, rigidity, and postural and gait disturbances are key in diagnosing PD, there are numerous non-motor symptoms (NMS) that associate with PD and add to its complexity in terms of patient experience and management. A wide range of interrelated cellular mechanisms are implicated in the pathogenesis of PD, including protein misfolding, mitochondrial dysfunction, oxidative stress, and neuroinflammation (Kalia and Lang, 2015). Search for disease-modifying treatments targeting putative pathogenic proteins has not been successful. Exercise has widespread symptom-related benefits in PD, improving motor and NMS of PD, and may be the only intervention available that can both slow disease progression and reduce the risk of developing PD (Sasco et al., 1992; Yang et al., 2015; Combs-Miller and Moore, 2019; Paul et al., 2019). While there are multiple mechanisms by which exercise may exert its benefits, here we describe the effects of exercise on neuroendocrine abnormalities associated with PD. We focus on a group of hormones – cortisol, melatonin, insulin, klotho, and vitamin D – specifically related to homeostasis and aging with relevance to PD.

## 2. Benefits of exercise in PD

The past decade has produced much evidence to support that exercise has neuroprotective benefits in PD (Zigmond et al., 2009; Tajiri et al., 2010; Tuon et al., 2012; Hou et al., 2017). Multiple mechanisms have been implicated including release of neurotrophic factors, improvements in mitochondrial function and oxidative stress, and anti-inflammatory effects (Tillerson et al., 2001; Cotman et al., 2007; Monteiro-Junior et al., 2015; Sujkowski et al., 2022). Acute exercise, or a single bout of exercise, initiates cellular pathways that transiently increase neurotransmitters, growth factors, and mediators of the “fight or flight” response, and increases cortical blood flow, oxygen availability, and glucose metabolism (El-Sayes et al., 2019). Regular exercise leads to chronic adaptations with upregulation of transcriptional and signaling cascades, cellular changes such as neurogenesis, synaptogenesis, gliogenesis and angiogenesis, as well as structural changes in the brain (El-Sayes et al., 2019). Studies in animal models of PD have shown that exercise ameliorates motor deterioration and loss of dopaminergic neurons (Tillerson et al., 2001; Petzinger et al., 2007; Zigmond et al., 2009).

In multiple prospective cohort studies, higher levels of physical activity have been associated with lower risk of developing PD (Sasco et al., 1992; Yang et al., 2015). Longitudinal cohort studies have also demonstrated that regular exercisers have a slower rate of change in motor, cognitive, and quality of life outcomes (Oguh et al., 2014; Combs-Miller and Moore, 2019; Paul et al., 2019). Amara et al. report that people with PD who have a higher score on Physical Activity Scale of the Elderly have a slower rate of progression of motor function decline, cognitive decline, and depression and anxiety (Amara et al., 2019). Despite these known benefits of exercise, the underlying mechanisms remain unclear. One important mechanism may be that exercise modulates hormones that maintain homeostasis and delay neuroendocrine aging.

## 3. Neuroendocrine abnormalities in PD and regulation by exercise

The neuroendocrine system encompasses both endocrine outputs from the nervous system, as well as peripheral hormones that act on the brain. It is a key system affected in PD that has important implications for pathogenesis and targets for disease modification. Since its primary function is to maintain homeostatic balance, PD-related abnormalities in the neuroendocrine system can lead to extensive downstream effects on stress, circadian rhythm, insulin resistance, and brain aging.

### 3.1. Cortisol

#### 3.1.1. Cortisol dysregulation in PD

The hypothalamic–pituitary–adrenal (HPA) axis is a pivotal neuroendocrine axis that modulates physiological homeostasis, stress responsivity and circadian function. The hypothalamus secretes corticotrophin-releasing hormone, which stimulates the anterior pituitary to release adrenocorticotropic hormone (ACTH). This ultimately signals the adrenal glands to release cortisol, which has widespread effects on the brain, affecting mood, behavior, cognition, and programming of the stress response (Viho et al., 2019).

Increasing evidence points to HPA axis dysregulation in PD. Heightened activity with elevated ACTH and cortisol levels has been reported in people with PD (Stypula et al., 1996). Cortisol levels also appear to be persistently elevated in blood (Hartmann et al., 1997; Breen et al., 2014; Costa et al., 2019) and saliva (Djamshidian et al., 2011; Skogar et al., 2011) of PD patients. Additionally, cortisol levels increase with aging and chronic stress, both of which are risk factors for PD (Lupien et al., 2009, 2018; Moffat et al., 2020). Epidemiological studies have revealed that higher job demands and expectations (Sieurin et al., 2018) and higher number of exposures to stressful events (Vlajinac et al., 2013) associate with an increased risk of development of PD.

Changes in stress and thus changes in cortisol can affect diverse symptoms of PD. Stress modulates motor system function since most parts of the motor system express glucocorticoid receptors, the primary receptor for cortisol (Metz, 2007). In PD mouse models, chronic stress exposure worsens motor deficits, aggravates neurodegeneration of the nigrostriatal system, and completely blocks compensatory recovery of motor tasks (Smith et al., 2008). In people newly diagnosed with PD, higher cortisol levels have been linked with greater deterioration in motor function (Haglin and Backman, 2016). Cortisol levels have also been associated with severity of depression (Seifried et al., 2013), prevalence of anxiety and anhedonia (van den Heuvel et al., 2020) and increased risk-taking in individuals with PD who have impulse compulsive behavior (Djamshidian et al., 2011).

#### 3.1.2. Acute and chronic effects of exercise on cortisol

Exercise acts both as a stressor and modifier of the neuroendocrine stress response (Hackney, 2006). Acute exercise, or single exercise session, activates the short-term stress response and increases salivary and plasma cortisol during the initial 30–60 min post-exercise (Wang et al., 2019; Dote-Montero et al., 2021). Moreover, Luger et al. have shown that this response appears to be directly proportional to the volume or intensity of exercise (Luger et al., 1987). An exercise intensity of 50–60% maximal oxygen uptake is the threshold before there is a change in cortisol, and this response amplifies when the intensity increases to 85%. In a study of elderly healthy men, cortisol released in response to acute vigorous exercise suppressed the subsequent cortisol response when presented with a psychosocial stressor, and this effect is dependent on exercise intensity (Caplin et al., 2021). Importantly, post-awakening salivary cortisol secretion and the cortical awakening response is significantly lower on the morning following an exercise session in healthy individuals (Anderson et al., 2023).

Chronic, or long-term, exercise training, defined as multiple sessions per week for duration ≥12 weeks in human studies, has different effects compared to acute exercise. Six months of high-intensity endurance exercise in women with mild cognitive impairment resulted in reduced plasma cortisol levels and improved executive cognitive function (Baker et al., 2010). A meta-analysis by Beserra et al. reports that in individuals with major depressive disorder, regular exercise is associated with decreased daytime cortisol levels but this is influenced by type of exercise and frequency (Beserra et al., 2018). Studies looking at the effects of exercise on cortisol levels in PD are sparse, but one study reports that 6 months of regular high-intensity treadmill exercise reduces cortisol secretion during the post-awakening period in people with PD (Smyth et al., 2019). Further studies are needed to clarify the acute and chronic effects of exercise on cortisol levels in individuals with PD and how this relates to motor and NMS of PD. Given initial studies showing an association between cortisol and mood, cognition, and motor burden in PD, it is possible that exercise-medicated regulation of cortisol may improve these symptoms.

### 3.2. Melatonin

#### 3.2.1. Melatonin and circadian rhythm disruption in PD

Another key function of the neuroendocrine system is regulation of the circadian rhythm, orchestrated by the central biological clock – the suprachiasmatic nucleus (SCN). SCN is located within the hypothalamus and is attuned to the environmental 24 h cycle by projections from the retinohypothalamic tract, oscillators in peripheral tissues, and circulating neurotransmitters and hormones such as serotonin, cortisol, and melatonin (Saper et al., 2005; Dibner et al., 2010; Blume et al., 2019). SCN neurons adjust their circadian phase according to these photic and non-photic inputs and signal using humoral and autonomic nervous systems to the rest of the body. A key output pathway of the SCN is its projection to the pineal gland where melatonin is produced (Cajochen et al., 2003). Therefore, these input and output pathways are reciprocal. The neurohormone melatonin acts as a transmission signal to coordinate and stabilize the circadian rhythm and regulate hormone secretion, core temperature, cognition, and mood (Cardinali, 2021).

Increasing evidence points to circadian rhythm disruption being a key feature in PD, one that influences not just symptom expression but potentially also PD-associated neurodegeneration. Reduced SCN firing rate has been reported in PD mouse models, which may lead to overall decreased circadian output (Kudo et al., 2011). Breen et al. have reported hypothalamic volume loss and associated decreased melatonin output in people with PD (Breen et al., 2016). A cross-sectional study also confirmed significantly diminished amplitude and amount of melatonin secretion in individuals with PD compared to controls, which associated with excessive daytime sleepiness but not motor function (Videnovic et al., 2014). In a large cohort of newly diagnosed PD, the majority of participants showed abnormal sleep macro-architecture including increased sleep latency, reduced sleep efficiency, and reduced REM sleep, associating with lower circulating melatonin levels (Breen et al., 2014).

Accumulating evidence shows that melatonin has anti-apoptotic, anti-oxidant, anti-inflammatory, and free radical-scavenging properties that protect against mitochondrial dysfunction (Leon et al., 2004; Rodriguez et al., 2004; Esposito and Cuzzocrea, 2010; Singhal et al., 2011, 2012). An additional mechanism by which melatonin may exert its benefit is through stimulating autophagy and inhibiting aggregation of prion-like proteins such as α-synuclein that misfold and aggregate in PD (Jeong et al., 2012). Evidence for improvements in motor function have been observed with administration of 3-10 mg/kg of melatonin in multiple studies using rodent models of PD (Kim et al., 1998; Sharma et al., 2006; Gutierrez-Valdez et al., 2012; Zaitone et al., 2013). On the other hand, a study by Bassani et al. suggests that while administration of melatonin 10 mg/kg protects against dopaminergic loss and improves depression, it does not improve motor deficits in rats (Bassani et al., 2014).

In individuals with mild cognitive impairment, treatment with 3-9 mg of melatonin for 2 years has been shown to improve cognitive and depressive symptoms in addition to sleep (Jean-Louis et al., 1998; Furio et al., 2007; Cardinali et al., 2012). While 3–9 mg of melatonin improves sleep qualities and rapid eye movement (REM) sleep behavior disorder (RBD; Takeuchi et al., 2001; Medeiros et al., 2007), studies have failed to show an improvement in motor symptoms (Medeiros et al., 2007; Liguori et al., 2022). RBD appears in the prodromal phase of PD, preceding motor symptoms of PD by several years (Boeve et al., 2001; Iranzo et al., 2006; Tolosa et al., 2007). Studies have reported that >80% of individuals who initially developed RBD had progressed to develop PD, related synucleinopathy, or dementia within 10 years (Iranzo et al., 2013; Schenck et al., 2013). An interesting case report has described increase in dopamine transport density (as assessed by dopamine transporter scintigraphy) in an elderly male patient with RBD treated with 2 mg of melatonin daily (Kunz and Bes, 2017). After 6 months of treatment, his RBD disappeared. His dopamine transporter scan with binding ratios in the abnormal range before treatment showed improved binding ratios in the normal range 2 years after treatment with melatonin and remained normal even 4 years after the first scan. Although a case report provides only one patient’s response to melatonin therapy, it suggests that melatonin may have neuroprotective benefits, warranting further investigations.

#### 3.2.2. Exercise regulates melatonin

There is growing interest in the use of exercise to synchronize the circadian rhythm, with studies showing that scheduled exercise can entrain the human sleep–wake rhythm. Vigorous wheel running exercise prevents the loss of rhythmicity induced from maintaining rats under constant light, and accelerates the emergence of a circadian pattern in rats moved to dim light (Lax et al., 1998). In healthy elderly men, a 3-month exercise training program induced a significant reduction in the fragmentation of their rest-phase rhythm (Van Someren et al., 1997). Exercise effects on improving the circadian rhythm clock are likely mediated by its effect on melatonin secretion. Exercise can have phase-shifting effects on melatonin secretion and lead to both acute and chronic increase in melatonin levels (Buxton et al., 1997). Exercise has also been shown to be beneficial in treatment of circadian rhythm disorders such as sleep, jet lag, and shift work disorder (Eastman et al., 1995; Shiota et al., 1996). Intensity and time of exercise also influences melatonin secretion, with melatonin levels increasing more with high-intensity exercise as compared to moderate-intensity exercise and morning exercise leading to a greater increase in melatonin at night compared to afternoon exercise (Kim and Kim, 2014; Carlson et al., 2019). Studies investigating whether exercise increases melatonin in people with PD are lacking. However, based on the known disruption in melatonin secretion in PD and the beneficial effects of melatonin administration in preclinical models of disease and in clinical trials of people with MCI and sleep disorders, exercise may be an essential way to restore melatonin levels and circadian rhythm in PD.

### 3.3. Insulin

#### 3.3.1. Insulin resistance and PD

It is now well recognized that there is an association between type II diabetes mellitus and increased risk of developing PD (Cullinane et al., 2022). (Figure 1) In the periphery, insulin regulates glucose metabolism, but within the central nervous system, it has neuromodulatory, neuroprotective, and neurotrophic effects. The majority of insulin comes from pancreatic B-cells, and is transported across the blood–brain barrier; however insulin and the closely related insulin-like growth factor 1 (IGF-1) are also produced by pyramidal neurons in the cortex, hippocampus, and olfactory bulb (Ghasemi et al., 2013). Insulin receptors are abundantly found in the basal ganglia and substantia nigra (Figlewicz et al., 2003).

Insulin regulates dopaminergic transmission, maintenance of synapses, synaptic plasticity, and neuronal survival and growth (Chen et al., 2022). A marked loss of insulin receptor mRNA in the substantia nigra pars compacta and increased levels of insulin receptor phosphorylation, which deactivates insulin signaling, has been reported in PD (Moroo et al., 1994; Takahashi et al., 1996; Sekar and Taghibiglou, 2018). Moroo et al. have shown that in PD brains *post mortem*, there is almost total loss of insulin receptor-positive neurons in the substantia nigra while some dopaminergic neurons remain, suggesting that downregulation in the insulin receptor system may precede the death of dopaminergic neurons (Moroo et al., 1994). Both insulin deficiency and insulin resistance lead to decreased brain insulin signaling in PD and contribute to neuroinflammation, mitochondrial dysfunction, and oxidative stress (Cullinane et al., 2022). Induction of insulin signaling with IGF-1 and reversal of insulin resistance suppresses α-synuclein aggregation and toxicity (Kao, 2009).

In individuals with PD, insulin resistance associates with a more severe phenotype, accelerated disease progression, and increased risk of cognitive decline (Cereda et al., 2012; Kotagal et al., 2013; Malek et al., 2016; Mohamed Ibrahim et al., 2018). Population-based cohort studies have also shown that use of certain antidiabetic drugs (glitazones, glucagon-like peptide-1 receptor agonists and dipeptidyl peptidase inhibitors), associate with a lower risk of developing PD (Brakedal et al., 2017; Brauer et al., 2020; Cullinane et al., 2022). The glucagon-like peptide-1 receptor agonist–exenatide–is also being explored as a potential disease modifying agent in PD (Cardoso and Moreira, 2020; Mulvaney et al., 2020).

#### 3.3.2. Exercise improves insulin sensitivity

Exercise has long been known to improve insulin sensitivity peripherally (Borghouts and Keizer, 2000; Sampath Kumar et al., 2019). In the brain, animal studies also point to exercise having a favorable impact on insulin signaling pathways (Park et al., 2005; Muller et al., 2011). In rodent models of insulin resistance and impairment in insulin receptor function in the hippocampus, treadmill exercise can rescue this decline in insulin signaling (Park et al., 2019). In rats with memory impairments, exercise raises insulin signaling in conjunction with improved cognitive function (Jeong et al., 2018).

In middle-aged sedentary individuals, exercise for 8 weeks was shown to increase brain insulin sensitivity following intranasal insulin administration (Kullmann et al., 2022). Honkala et al. demonstrated that sprint interval training, but not moderate-intensity continuous training, decreased insulin-stimulated glucose uptake in cortical gray matter and most brain regions in insulin-resistant, middle-aged adults (Honkala et al., 2018). Exercise also influences IGF-1 gene expression and protein levels in several brain regions, especially those involved in learning and cognition (Duzel et al., 2016). Exercise has been shown to induce increase in circulating IGF-1 in the periphery which can be transported through the blood–brain barrier into the brain (Schwarz et al., 1996). Further studies need to explore the effects of exercise on insulin sensitivity and IGF-1 levels in PD. Exercise-enhanced insulin signaling may be crucial in PD to counter the detrimental downstream effects of insulin resistance, such as neuroinflammation, mitochondrial dysfunction, and oxidative stress and a more severe PD phenotype.

### 3.4. Klotho

#### 3.4.1. A role for longevity hormone klotho in PD

Klotho was discovered as a master aging regulator when deficiency of the klotho protein in mice was found to severely shorten lifespan and prompt signs of premature aging while overexpression of klotho increased lifespan by ~30% (Kurosu et al., 2005; Zeldich et al., 2014). Additionally, klotho-insufficient mice develop degeneration of mesencephalic dopaminergic neurons in substantia nigra and ventral tegmentum area (Kosakai et al., 2011). Human relevance for klotho in longevity is supported by studies demonstrating that klotho levels decline with aging (Yamazaki et al., 2010; Semba et al., 2011) and klotho genetic variants are associated with lifespan in multiple aging populations (Arking et al., 2002, 2005). Mechanistically, klotho has multifactorial actions–it suppresses insulin and *Wnt* signaling (Utsugi et al., 2000; Liu et al., 2007), regulates ion channel clustering and transport (Chang et al., 2005), modulates *N*-methyl-d-aspartate receptor (NMDAR) signaling (Dubal et al., 2014) and promotes fibroblast growth factor (FGF) function (Urakawa et al., 2006). Klotho linked with FGF23 is important for regulation of calcium, phosphate, and vitamin D homeostasis (Tsujikawa et al., 2003; Razzaque, 2009; Huang and Moe, 2011; Hum et al., 2017; Erben, 2018; Kuro, 2018). Klotho overexpression also inhibits insulin/IGF-1 signaling as a mechanism to extend life (Kurosu et al., 2005). While insulin resistance increases with aging and thus is generally unfavorable, klotho’s role in inhibiting the insulin/IGF-1 pathway is to decrease lipid overload since lipid-laden cells are vulnerable to lipid-induced programmed cell death (Unger, 2006).

Mounting evidence now links klotho with enhanced cognition. In mouse models of aging and Alzheimer’s disease, systemic elevation of klotho boosts cognitive function and enhances long-term potentiation (Dubal et al., 2014, 2015; Masso et al., 2018; Zeng et al., 2019). Recently, subcutaneous delivery of low but not high dose klotho was shown to enhance cognition in aged rhesus monkeys (Castner et al., 2023). In humans, carrying a genetic variant of *KLOTHO* or having higher levels of klotho has been associated with better cognition or decreased risk of dementia in aging and in Alzheimer’s disease (Dubal et al., 2014; Yokoyama et al., 2015; Belloy et al., 2020; Ali et al., 2022; Kundu et al., 2022).

In PD mouse models, peripherally delivered klotho has been shown to result in improvement in cognitive and motor behavior and enhanced synaptic plasticity (Leon et al., 2017). Furthermore, klotho overexpression or exogenous klotho administration protects dopaminergic neurons against oxidative injury and alleviates astrogliosis, apoptosis, and oxidative stress (Baluchnejadmojarad et al., 2017; Leon et al., 2017). Recently, lower levels of cerebrospinal fluid klotho were reported in individuals with PD compared to healthy controls and associated with greater motor burden of disease and greater Hoehn and Yahr stage of disability (Zimmermann et al., 2021). Since aging is a major risk factor for PD, further studies are needed to investigate if klotho associates with clinical symptoms of PD or disease progression.

#### 3.4.2. Exercise upregulates klotho

Given the known beneficial effects of exercise on health and longevity, it is not surprising that exercise is associated with changes in aging regulator klotho. Higher levels of klotho associate with superior lower extremity strength and functioning in older adults (Crasto et al., 2012; Semba et al., 2016; Saghiv et al., 2017). Klotho levels also tend to be higher in exercise-trained individuals compared to their untrained counterparts (Saghiv et al., 2017). In healthy middle-aged adults, endurance, or resistance exercise boosts plasma klotho levels both acutely and chronically (Matsubara et al., 2014; Santos-Dias et al., 2017; Tan et al., 2018; Amaro-Gahete et al., 2019). When looking at the underlying mechanisms of exercise, Jin et al. demonstrated that irisin, an exercise-derived myokine, enhances mortality and cognition in mice after cerebral ischemia through upregulation of klotho (Jin et al., 2021). This positive effect of exercise and irisin is abolished in klotho-knockout mice (Jin et al., 2021). Exercise-induced upregulation of klotho in rodents has also been linked to decreased production of reactive oxygen species leading to decreased oxidative stress (Ji et al., 2018).

Additional studies are needed to examine the effects of exercise on klotho in people with PD. With increasing evidence for klotho’s impact on longevity, cognition, and neuroprotection, there is fervent interest in development of klotho-boosting therapeutics. Exercise may be one way to enhance klotho levels that may help with PD symptoms and progression.

### 3.5. Vitamin D

#### 3.5.1. Multifunctional hormone vitamin D in PD

Vitamin D is a powerful neurosteroid that can pass the blood–brain barrier to play a significant role in the central nervous system. While in the periphery vitamin D is critical for bone metabolism and calcium regulation, its effects in the brain are vast. It plays a role in anti-inflammatory and anti-oxidant actions, intracellular calcium signaling, neurotransmitter release, and transcriptional regulation of >200 genes, including neurotrophins (Pignolo et al., 2022).

Vitamin D receptor concentration is richest in the substantia nigra and the hypothalamus (Eyles et al., 2005). Additionally, 1α-hydroxylase – the enzyme that converts vitamin D to its active form,1,25(OH)_2_D_3_ – is highly expressed in the substantia nigra, suggesting that vitamin D may be related to the pathogenesis of PD via loss of protection for vulnerable dopaminergic neurons in this brain region (Eyles et al., 2005). In their meta-analysis, Zhou et al. report that both 25-hydroxyvitamin D [25(OH)D] insufficiency (<30 ng/ml) and deficiency (<20 ng/mL) are significantly associated with an increased risk of PD (Zhou et al., 2019). A high prevalence (55–69%) of 25(OH)D deficiency has been noted in individuals with PD (Evatt et al., 2008, 2011). Higher vitamin D concentrations have also been shown to associate with better cognitive function and mood in individuals with PD (Peterson et al., 2013).

Peterson et al. demonstrate that treatment with vitamin D (30 mg/kg subcutaneously) in a rat model of PD attenuates behavioral deficits, decreases levels of oxidative stress and neuroinflammatory markers and prevents dopaminergic cell loss (Bayo-Olugbami et al., 2022). Administration of intraperitoneal vitamin D (1 μg/ml/kg per day) in rats has also been shown to reduce dopaminergic cell loss and increase expression of glial-derived neurotrophic factor (Sanchez et al., 2009). In a randomized, placebo-controlled trial, vitamin D supplementation at a dose of 1,200 international units per day for 12 months in PD participants with a certain vitamin D receptor genotype (Fok/CC) associated with better motor function, Hoehn & Yahr stage, and quality of life (Suzuki et al., 2013). Interestingly, the Fok/CC vitamin D receptor genotype has also previously been shown to be associated with less advanced cases of PD (Suzuki et al., 2012) and to have greater effects of vitamin D-dependent transcriptional activation as compared to the Fok/TT genotype. In another recent randomized placebo-controlled trial, Bytowoska et al. investigated the effects of vitamin D ranging in dose from 2,500 to 4,800 international units per day (based on body mass index) in patients with PD who had previously undergone deep brain stimulation surgery (Bytowska et al., 2023). They reported significant improvements in tests of ambulatory function and a downward trend, though not significant, in inflammatory marker C-reactive protein, in the group that received vitamin D supplementation for 12 weeks. As demonstrated in a recent review, C-reactive protein is a robust marker of inflammation in PD (Mehta et al., 2023). Given the potential benefits of vitamin D, improving vitamin D status in individuals with PD may be a valuable approach.

#### 3.5.2. Vitamin D response to exercise

Vitamin D levels increase acutely after exercise, and these changes are further dependent on intensity of exercise, age, and sex (Maimoun et al., 2009; Sun et al., 2017; Dzik et al., 2022). In their meta-analysis, Zhang and Cao report that in most studies with chronic exercise (multiple sessions per week for >12 weeks), there is an increase in 25(OH)D in vitamin D-deficient individuals but not in vitamin-D sufficient individuals (Zhang and Cao, 2022). This positive effect has been more commonly reported in studies of endurance exercise, possibly due to lipolytic processes leading to release of vitamin D stored in adipose tissues. Exercise can also increase vitamin D receptor mRNA levels (Aly et al., 2016; Puangthong et al., 2021). Da Costa et al. report that vitamin D receptor expression is decreased in hemiparkinsonian rats, but this effect is attenuated with treadmill exercise (da Costa et al., 2022). Furthermore, they demonstrated that administering vitamin D3 with exercise improved behavioral outcomes, augmented dopamine levels, and rescued dopaminergic neurons as compared to exercise alone.

## 4. Discussion

We have selected and discussed a group of five hormones with links to PD and have highlighted their role in maintaining homeostasis and longevity and their responsiveness to exercise. These documented neuroendocrine abnormalities are important for several reasons: (1) increased or decreased levels of these hormones associate with various NMS of PD, such as mood, cognition, and sleep/circadian rhythm disorder in addition to classic motor symptoms, (2) these hormones act on pathways implicated in PD pathogenesis, including neuroinflammation, oxidative stress, and neurodegeneration, (3) these hormones may provide a mechanistic pathway for exercise-induced neuromodulatory effects and (4) these hormones may represent important biomarkers to measure exercise effects in clinical trials. Future studies are needed to confirm that these hormones respond to exercise specifically in individuals with PD and affect symptoms or disease progression. These insights might also help people with PD to feel empowered that they have some control over their disease if provided with evidence that regulation of these hormones has an impact on their condition (Table 1).

**Table 1:** Exercise effects on neuroendocrine targets.

Hormone	Response to exercise	References
Cortisol	Increased with acute exercise; decreases with long-term exercise	Luger et al. (1987), Hackney (2006), Baker et al. (2010), Beserra et al. (2018), Smyth et al. (2019), Wang et al. (2019), Caplin et al. (2021), Dote-Montero et al. (2021) and Anderson et al. (2023)
Melatonin	Increased levels	Eastman et al. (1995), Shiota et al. (1996), Buxton et al. (1997), Lax et al. (1998), Kim and Kim (2014) and Carlson et al. (2019)
Insulin	Improved insulin sensitivity; increased IGF-1	Schwarz et al. (1996), Borghouts and Keizer (2000), Park et al. (2005), Muller et al. (2011), Duzel et al. (2016), Honkala et al. (2018), Jeong et al. (2018), Park et al. (2019), Sampath Kumar et al. (2019) and Kullmann et al. (2022)
Klotho	Increased levels	Matsubara et al. (2014), Saghiv et al. (2017), Santos-Dias et al. (2017), Ji et al. (2018), Tan et al. (2018), Amaro-Gahete et al. (2019), Amaro-Gahete et al. (2019) and Jin et al. (2021)
Vitamin D	Increased levels; increased VDR expression	Maimoun et al. (2009), Aly et al. (2016), Sun et al. (2017), Puangthong et al. (2021), da Costa et al. (2022), Dzik et al. (2022) and Zhang and Cao (2022)

IGF-1, Insulin-like growth factor I; VDR, vitamin D receptor.

While there is a general consensus that exercise has vast benefits and should be recommended to people, the type and intensity of the exercise prescription is not always clear. Measuring biomarkers, such as the neuroendocrine targets described here, can provide objective data to compare the effects of different modalities, frequencies, and intensities of exercise. The effects of exercise may also vary with disease stage or subtype of PD and these variables need to be considered in future clinical trials of exercise in PD.

This perspective is limited in that it reviews the effects of exercise on PD-associated neuroendocrine abnormalities linked to aging and stress. However, there are other hormone imbalances in PD that have been reported and have been discussed elsewhere: (1) ghrelin and leptin that together regulate feeding behavior (Fiszer et al., 2010; Unger et al., 2011; Bayliss and Andrews, 2013), (2) growth hormone, luteinizing hormone, and estrogen that may explain mechanisms underlying sex differences in PD (Bonuccelli et al., 1990; Crespi, 1993; Ragonese et al., 2007; Hirohata et al., 2009; Georgiev et al., 2017; Vaidya et al., 2021), and (3) emerging hormones such as irisin, which can cross the blood brain barrier and has been shown to reduce pathological α-synuclein and rescue dopaminergic neurons from degeneration (Zarbakhsh et al., 2019; Kam et al., 2022; Zhang et al., 2023). Lastly, while neuroendocrine is one system that is affected in PD and responds to exercise, it is beyond the scope of this review to discuss other important systems such as neurotrophic factors and inflammatory markers that also respond robustly to exercise.

There is strong evidence-based rationale for the recommendation of regular exercise as a symptom-modifying, and possibly disease-attenuating, treatment in PD. Evolving data further supports exercise-induced changes in biomarkers but additional clinical studies are needed to link changes in these biomarkers to observed benefits in people with PD.

## 5. Conclusion

We conclude that exercise leads to neuroendocrine normalization with potential neuroprotective effects relevant to PD. Collectively these hormones are involved in critical functions such as maintaining homesostasis and responding to stress, sleep and circadian rhythm, insulin sensitivity, longevity, and brain health. Instead of targeting each neuroendocrine abnormality specifically, exercise provides an advantage in that it can regulate multiple hormones and thus lead to improvement in motor and non-motor symptoms and potentially slow progression of disease.

## Data availability statement

The original contributions presented in the study are included in the article/supplementary material, further inquiries can be directed to the corresponding author.

## Author contributions

NL: design, execution, writing, and editing of the final version of the manuscript. DCh: writing and editing of the final version of the manuscript. AC: execution, writing, and editing of the final version of the manuscript. DCo: design, execution, and editing of the final version of the manuscript. All authors contributed to the article and approved the submitted version.

## Funding

Research reported in this publication was supported by the National Institute of Neurological Disorders and Stroke of the National Institutes of Health under Award Numbers U01NS113851 and K23NS123506. Research is also supported by the National Institutes of Health’s National Center for Advancing Translational Sciences, Grant Number UL1TR001422. The content is solely the responsibility of the authors and does not necessarily represent the official views of the National Institutes of Health. The research is also supported by a generous philanthropic gift in honor of Howard Gilbert.

## Conflict of interest

The authors declare that the research was conducted in the absence of any commercial or financial relationships that could be construed as a potential conflict of interest.

## Publisher’s note

All claims expressed in this article are solely those of the authors and do not necessarily represent those of their affiliated organizations, or those of the publisher, the editors and the reviewers. Any product that may be evaluated in this article, or claim that may be made by its manufacturer, is not guaranteed or endorsed by the publisher.
